# RETRACTION: Complications and Psychological Impact of Pressure Ulcers on Patients and Caregivers

**DOI:** 10.1111/iwj.70547

**Published:** 2025-04-18

## Abstract

**RETRACTION:** QianL. , YanS. , TingS. T. , HanZ. M. , and QiT. , “Complications and Psychological Impact of Pressure Ulcers on Patients and Caregivers,” International Wound Journal 21, no. 4 (2024): e14836, 10.1111/iwj.14836.38531386 PMC10965270

The above article, published online on 26 March 2024, in Wiley Online Library (http://onlinelibrary.wiley.com/), has been retracted by agreement between the journal Editor in Chief, Professor Keith Harding; and John Wiley & Sons Ltd. Following an investigation by the publisher, all parties have concluded that this article was accepted solely on the basis of a compromised peer review process. The editors have therefore decided to retract the article. The authors did not respond to our notice regarding the retraction.

**RETRACTION:** L. Qian , S. Yan , S. T. Ting , Z. M. Han , and T. Qi , “Complications and Psychological Impact of Pressure Ulcers on Patients and Caregivers,” International Wound Journal 21, no. 4 (2024): e14836, 10.1111/iwj.14836.38531386 PMC10965270

The above article, published online on 26 March 2024, in Wiley Online Library (http://onlinelibrary.wiley.com/), has been retracted by agreement between the journal Editor in Chief, Professor Keith Harding; and John Wiley & Sons Ltd. Following an investigation by the publisher, all parties have concluded that this article was accepted solely on the basis of a compromised peer review process. The editors have therefore decided to retract the article. The authors did not respond to our notice regarding the retraction.

